# Effect of Minor P and C Regulation on Microstructure Evolution and High-Temperature Properties of Selective Laser Melted GH4169 Superalloy

**DOI:** 10.3390/ma19183910

**Published:** 2026-09-15

**Authors:** Wenhan Wang, Ang Li, Zhaopeng Hou, Yunwei Gui, Bingtao Li, Hongyao Yu, Guohao Liu, Huadong Fu

**Affiliations:** 1 School of Materials Science and Engineering, University of Science and Technology Beijing, Beijing 100083, China; 2 Gaona Aero Material Co., Ltd., Beijing 100081, China; 3 Research Institute of Advanced Materials (Shenzhen) Co., Ltd., Shenzhen 518045, China; 4 Beijing Advanced Innovation Center for Materials Genome Engineering, School of Advanced Materials Innovation, University of Science and Technology Beijing, Beijing 100083, China

**Keywords:** selective laser melting, GH4169 superalloy, minor P and C additions, phase evolution, high-temperature tensile behavior

## Abstract

Selective laser melting (SLM) produces elemental segregation and nonequilibrium secondary phases in GH4169 superalloys, but the effects of P and C variations on grain-boundary phase evolution and high-temperature tensile behavior remain unclear. Alloys with primarily varied P (0.034–0.058 wt.%) and C (0.009–0.066 wt.%) contents were fabricated and heat treated identically. Microstructures were characterized by scanning electron microscopy (SEM), electron backscatter diffraction (EBSD), and transmission electron microscopy (TEM), and tensile properties were evaluated at 650 °C. The as-built alloys exhibited columnar grains, cellular substructures, cell-wall segregation, and Nb-rich Laves-phase particles. After heat treatment, cellular substructures largely disappeared, γ″ precipitates formed in the γ matrix, and fine Ti/Al-rich particles were tentatively attributed to γ′; δ phase, residual Laves phase, and MC-type carbides were present at grain boundaries. Increasing P reduced the δ-phase area fraction from 2.20% to 1.38% and changed its distribution from continuous chains to semi-continuous and discrete arrangements. Increasing C raised the MC-type carbide area fraction from 0.34% to 1.48% while decreasing the δ-phase area fraction from 1.68% to 0.98%, accompanied by carbide coarsening. The 0.050P alloy exhibited the highest yield and ultimate tensile strengths, whereas the 0.009C alloy showed the best strength–ductility balance. These results reveal distinct P- and C-related changes in grain-boundary phase evolution and tensile behavior at 650 °C.

## 1. Introduction

GH4169 is a typical precipitation-strengthened Ni-based superalloy in which the γ″-Ni_3_Nb phase serves as the primary strengthening phase [1]. It exhibits excellent high-temperature strength, oxidation resistance, corrosion resistance, processability, and weldability and has therefore been widely used in aero-engine turbine disks, casings, fasteners, and other high-temperature load-bearing components [2,3,4]. As aerospace equipment continues to develop toward higher thrust-to-weight ratios, lightweight design, and structural integration, conventional casting, forging, and machining methods are increasingly confronted with challenges such as long manufacturing cycles, low material utilization, and considerable processing difficulty in the fabrication of complex internal channels, thin-walled structures, and multiscale components. As a representative metal additive manufacturing technology, selective laser melting (SLM) enables near-net-shape fabrication by selectively melting metal powder layer by layer using a high-energy laser beam. Its high degree of design freedom, high material utilization, and capability for fabricating complex structures provide a new technical route for the integrated manufacturing of complex GH4169 alloy components [2,3,4,5].

However, during SLM, metal powder undergoes rapid melting and solidification as well as repeated thermal cycling. The thermal gradient (G), solidification-front rate (R), and cooling rate were not measured under the present processing conditions. For context, published modeling studies relevant to IN718 SLM solidification have reported representative values of G = 2.6–4.6 × 10^6^ K·m^−1^ and R ≈ 0.1 m·s^−1^, while independent microstructure- and heat-transfer-based estimates place cooling rates broadly in the 10^5^–10^7^ K·s^−1^ range depending on processing conditions [6,7]. These values are cited only as literature-scale references and should not be interpreted as measurements for the present 300 W/960 mm·s^−1^ SLM process. Owing to directional heat conduction and epitaxial growth, as-built GH4169 alloys fabricated by SLM frequently exhibit columnar grains extending along the build direction, crystallographic texture, and fine cellular/dendritic substructures [4,5,6,7,8]. Meanwhile, rapid nonequilibrium solidification results in pronounced segregation of alloying elements such as Nb, Mo, and Ti at cell walls or within interdendritic regions and promotes the formation of Nb-rich Laves phase and MC-type carbides [3,8,9,10]. These Nb-rich secondary phases not only consume the Nb required for the formation of the γ″ strengthening phase, but their brittle nature and nonuniform distribution also tend to induce local stress concentrations, thereby affecting microstructural evolution during subsequent heat treatment and the resulting mechanical properties [9,10]. Therefore, SLM-fabricated GH4169 alloys generally require post-processing treatments, such as solution treatment and aging, to promote elemental homogenization and obtain γ′/γ″ strengthening precipitates dispersed within the γ matrix, while additional hot isostatic pressing may be employed when necessary to reduce internal porosity and residual defects [11,12,13,14].

During the heat treatment of GH4169 alloys, the redistribution of Nb among the γ matrix, Laves phase, δ phase, γ″ phase, and MC-type carbides is a key factor governing microstructural stability and high-temperature mechanical properties [9,10,12]. The γ″ phase is a Ni_3_Nb phase with an ordered D0_22_ structure and is the primary strengthening phase in GH4169 alloys under service conditions near 650 °C. The γ′ phase, Ni_3_(Al,Ti), can further improve the matrix strength through its synergistic effect with the γ″ phase [1,11,14]. However, γ″ is metastable, and prolonged thermal exposure, particularly at temperatures above its stability range, can promote its transformation into the thermodynamically stable δ phase [1,14,15]. The δ phase is also an Nb-rich Ni_3_Nb phase but has an orthorhombic D0a structure and generally precipitates preferentially at grain boundaries and in Nb-rich regions [9,14,15]. An appropriate amount of discontinuously distributed δ phase can effectively pin grain boundaries and suppress grain-boundary migration at elevated temperatures, whereas coarse and continuously distributed δ phase consumes Nb from the matrix, reduces the strengthening potential of γ″, and provides preferential sites for cavity formation and intergranular crack propagation [15]. In addition, MC-type carbides possess relatively high thermal stability. Fine and uniformly distributed MC-type carbide particles can improve grain-boundary stability, whereas coarsened or agglomerated carbides may cause local deformation incompatibility and serve as crack-initiation sites during high-temperature deformation [15,16,17]. Therefore, controlling the amount, morphology, and spatial distribution of Nb-containing secondary phases is essential for optimizing the high-temperature properties of SLM-fabricated GH4169 alloys.

Although conventional heat treatments can regulate secondary-phase evolution to a certain extent, precise control of grain-boundary secondary phases remains difficult to achieve solely by optimizing heat-treatment parameters because of the inherent elemental segregation and nonequilibrium microstructural characteristics associated with the SLM process. In contrast, regulating elemental segregation behavior, interfacial stability, and secondary-phase precipitation through trace-element design provides a new strategy for improving the microstructure and properties of SLM-fabricated GH4169 alloys. Among these elements, P and C are key trace elements affecting grain-boundary behavior and the stability of Nb-related phases in GH4169 alloys and are therefore of considerable research interest. P has a strong tendency to segregate to grain boundaries in GH4169 alloys and can become enriched at grain boundaries and δ/γ interfaces. By altering the local interfacial energy, elemental diffusion behavior, and nucleation conditions of the δ phase, P can influence grain-boundary microstructural evolution. Previous studies have shown that an appropriate P content can improve grain-boundary stability and enhance the high-temperature stress-rupture and creep properties of the alloy. However, excessive P segregation may weaken grain-boundary cohesion and increase the tendency toward intergranular damage [18,19,20,21,22]. Notably, previous studies have mainly focused on conventionally cast and wrought GH4169 alloys, whereas the nonequilibrium segregation state formed during rapid solidification in SLM may alter the manner in which P affects microstructural evolution. Therefore, the segregation behavior of P in SLM-fabricated GH4169 alloys and the mechanism by which it regulates the precipitation morphology of the δ phase and grain-boundary stability remain insufficiently understood.

C mainly affects the microstructure and properties of GH4169 alloys by promoting the formation of MC-type carbides. C preferentially combines with strong carbide-forming elements such as Nb and Ti to form MC-type carbides predominantly containing Nb and a certain amount of Ti [16,17]. An appropriate amount of fine and dispersed MC-type carbides can improve grain-boundary stability, whereas a higher C content may promote carbide coarsening and agglomeration, which may lead to deformation incompatibility and crack initiation [15,16,17]. More importantly, both MC-type carbides and the δ phase consume Nb. Therefore, variations in C content may alter the competition between MC-type carbide formation and δ-phase precipitation, further affecting the effective Nb content in the γ matrix, the formation of the γ″ strengthening phase, and grain-boundary microstructural stability. However, studies concerning the regulation of competitive MC/δ precipitation by C and its influence on the high-temperature deformation response of SLM-fabricated GH4169 alloys remain limited.

Accordingly, this study characterizes the representative as-built microstructure and systematically investigates the effects of P and C contents on secondary-phase evolution in the heat-treated condition, and tensile properties at 650 °C in SLM-fabricated GH4169 alloys. Particular emphasis is placed on revealing the relationship between P segregation behavior and the precipitation characteristics of the δ phase, clarifying the mechanism by which C regulates MC-type carbide formation and the competitive precipitation of MC-type carbides and the δ phase, and establishing the relationships among trace-element content, grain-boundary secondary-phase evolution, and high-temperature mechanical properties. This study aims to deepen the understanding of trace-element regulation mechanisms in SLM-fabricated GH4169 alloys and to provide a theoretical basis for compositional design, microstructural optimization, and the improvement in the high-temperature service performance of additively manufactured Ni-based superalloys.

## 2. Materials and Methods

### 2.1. Materials and Specimen Preparation

The GH4169 pre-alloyed powder used in this study was commercially supplied by Gaona Aero Material Co., Ltd. (Beijing, China) and was produced by conventional gas atomization. Two compositional series were established to primarily vary P and C. The measured P/C contents (wt.%) were 0.034/0.035, 0.050/0.034, and 0.058/0.031 for the P series, and 0.057/0.009, 0.054/0.040, and 0.056/0.066 for the C series. The corresponding specimens were designated as 0.034P, 0.050P, 0.058P, 0.009C, 0.040C, and 0.066C, respectively. P and C were determined by inductively coupled plasma atomic emission spectroscopy (ICP-AES) and high-frequency combustion–infrared absorption, respectively. Specimens of all compositions were fabricated using an A300 SLM system (Xi’an Bright Laser Technologies Co., Ltd., Xi’an, China) with a laser power of 300 W, a scan speed of 960 mm·s^−1^, a hatch spacing of 0.11 mm, and a layer thickness of 40 μm. The scan direction was rotated by 67° between adjacent layers to reduce residual stress and microstructural anisotropy. Fabrication was conducted under a high-purity argon atmosphere, and the substrate preheating temperature was set to 80 °C. Following SLM fabrication, all specimens were subjected to hot isostatic pressing at 1175 °C and 160 MPa for 3 h followed by furnace cooling, solution treatment at 980 °C for 1 h followed by air cooling (AC), and aging at 720 °C for 8 h, followed by cooling to 620 °C, holding for 8 h, and air cooling.

### 2.2. Microstructural Characterization

All specimens used for microstructural characterization were extracted from the central region of the build. For each composition, one 10 × 10 × 10 mm^3^ specimen was used for microstructural characterization, and the electron backscatter diffraction (EBSD) reference direction was the build direction (BD). Metallographic specimens were etched for approximately 45 s using a solution containing 5 g CuCl_2_, 100 mL HCl, and 100 mL ethanol, followed by ethanol cleaning. Scanning electron microscopy (SEM) and energy-dispersive X-ray spectroscopy (EDS) observations were performed using a Thermo Scientific Apreo 2S field-emission scanning electron microscope(Thermo Fisher Scientific, Waltham, MA, USA) at an accelerating voltage of 20 kV and a beam current of 6.4 nA. EDS, including point analyses, line scans, and elemental mapping, was used for local microchemical characterization of the matrix and secondary-phase regions. ImageJ version 1.54g (National Institutes of Health, Bethesda, MD, USA) was used to segment the δ phase and MC-type carbides in backscattered-electron SEM images of the heat-treated specimens and to determine their two-dimensional area fractions. For each composition, five non-overlapping backscattered-electron (BSE) SEM fields of view were randomly selected at a nominal magnification of 5000× for statistical analysis. All images were acquired under consistent imaging conditions, and the final results were reported as mean values. The feature categories were distinguished using consistent BSE morphology/contrast together with SEM-EDS compositional characteristics, while transmission electron microscopy (TEM) and selected-area electron diffraction (SAED) provided independent confirmation of the phase types present. Crystallographic characterization was performed using the same Thermo Scientific Apreo 2S system equipped with an EBSD detector. EBSD data were acquired at 10 kV with a probe current of 3.2 nA and were processed using AZtec Crystal 2.1 (build 2.1.259, Oxford Instruments, Abingdon, UK). The inverse pole figure (IPF) maps were used to visualize grain morphology and crystallographic orientation relative to BD, and kernel average misorientation (KAM) maps were used to assess local misorientation. Accordingly, the present IPF analysis is interpreted qualitatively in terms of grain morphology and orientation relative to BD, without assigning a specific preferred crystallographic direction from the IPF colors alone.

An FEI Talos F200X transmission electron microscope (FEI Company, Hillsboro, OR, USA; TEM) was used for detailed characterization of the cellular substructures in the as-built condition and the precipitated phases in the heat-treated condition. TEM foils were prepared by electrochemical twin-jet thinning. Disks 3 mm in diameter and approximately 0.2 mm thick were prepared by wire cutting and mechanically ground to approximately 40 μm. Final electropolishing was performed at −30 °C in an 8 vol.% perchloric acid in ethanol at 25 V for 1–2 min, followed by ethanol cleaning. TEM observations were conducted at 200 kV. Bright-field (BF) imaging, dark-field (DF) imaging, selected-area electron diffraction (SAED), and high-resolution transmission electron microscopy (HRTEM) were employed to characterize precipitate morphologies and obtain crystallographic evidence. High-angle annular dark-field scanning transmission electron microscopy (HAADF-STEM), together with STEM-EDS point analyses, line scans, and elemental mapping, was used to determine local elemental distributions and compositions. γ″ identification was based on diffraction evidence together with morphology and compositional information; the δ phase and the Nb-rich MC-type carbide were identified using crystallographic/diffraction evidence together with morphology and STEM-EDS. Fine Ti/Al-rich particles without equivalent crystallographic confirmation are described as tentatively identified γ′. The γ″ precipitate size was measured from the representative TEM dark-field image using the major-axis length of the disk-shaped γ″ precipitates as the characteristic dimension.

X-ray diffraction (XRD) was performed on a representative as-built sample and a representative heat-treated sample using Rigaku SmartLabXE and SmartLab diffractometers (Rigaku Corporation, Tokyo, Japan), respectively, with Cu Kα radiation. The representative XRD patterns are displayed over 30–100° for qualitative phase comparison.

### 2.3. High-Temperature Tensile Testing

The alloys in the heat-treated condition were machined into flat tensile specimens with a dog-bone geometry using wire-cut electrical discharge machining (wire-cut EDM). For each composition, three high-temperature tensile specimens were tested. The tensile blanks had dimensions of 65 × 20 × 10 mm^3^, were prepared by wire-cut EDM, and were oriented with the tensile axis parallel to the build direction (BD). High-temperature tensile tests were conducted at 650 °C. This temperature was selected because it is representative of the elevated-temperature service regime of GH4169/IN718, in which γ″ remains the primary strengthening phase and the alloy is widely used for high-temperature load-bearing components [1,11,14]; tensile testing at this temperature has also been adopted in previous studies of SLM-fabricated IN718 [15]. After the specimens were heated to the target temperature, they were held for 15 min to ensure a stable temperature within the gauge section. Uniaxial tensile testing was then performed at a crosshead displacement rate of 2 mm·min^−1^ until complete fracture. The load and displacement data were continuously recorded, and the engineering stress–engineering strain curves were calculated using the initial cross-sectional area and gauge length of each specimen. Using a consistent data-processing method, the yield strength, ultimate tensile strength, and elongation to fracture were determined. After tensile fracture, the fracture surfaces were examined using the same Thermo Scientific Apreo 2S field-emission scanning electron microscope. Overview fracture-surface SEM images (nominal magnification 300×; 100 μm scale bars) were used to analyze the overall fracture morphology and the distributions of different fracture regions, whereas higher-magnification local observations were used to characterize microscopic fracture features, including dimples, tearing ridges, intergranular fracture regions, secondary-phase particles, and secondary cracks. The mechanical-property data were correlated with the fracture morphologies to assess how P- and C-related changes in grain-boundary secondary-phase evolution influenced high-temperature deformation and fracture behavior.

## 3. Results

### 3.1. Microstructural Characteristics of the As-Built GH4169 Alloy

**Figure 1:**
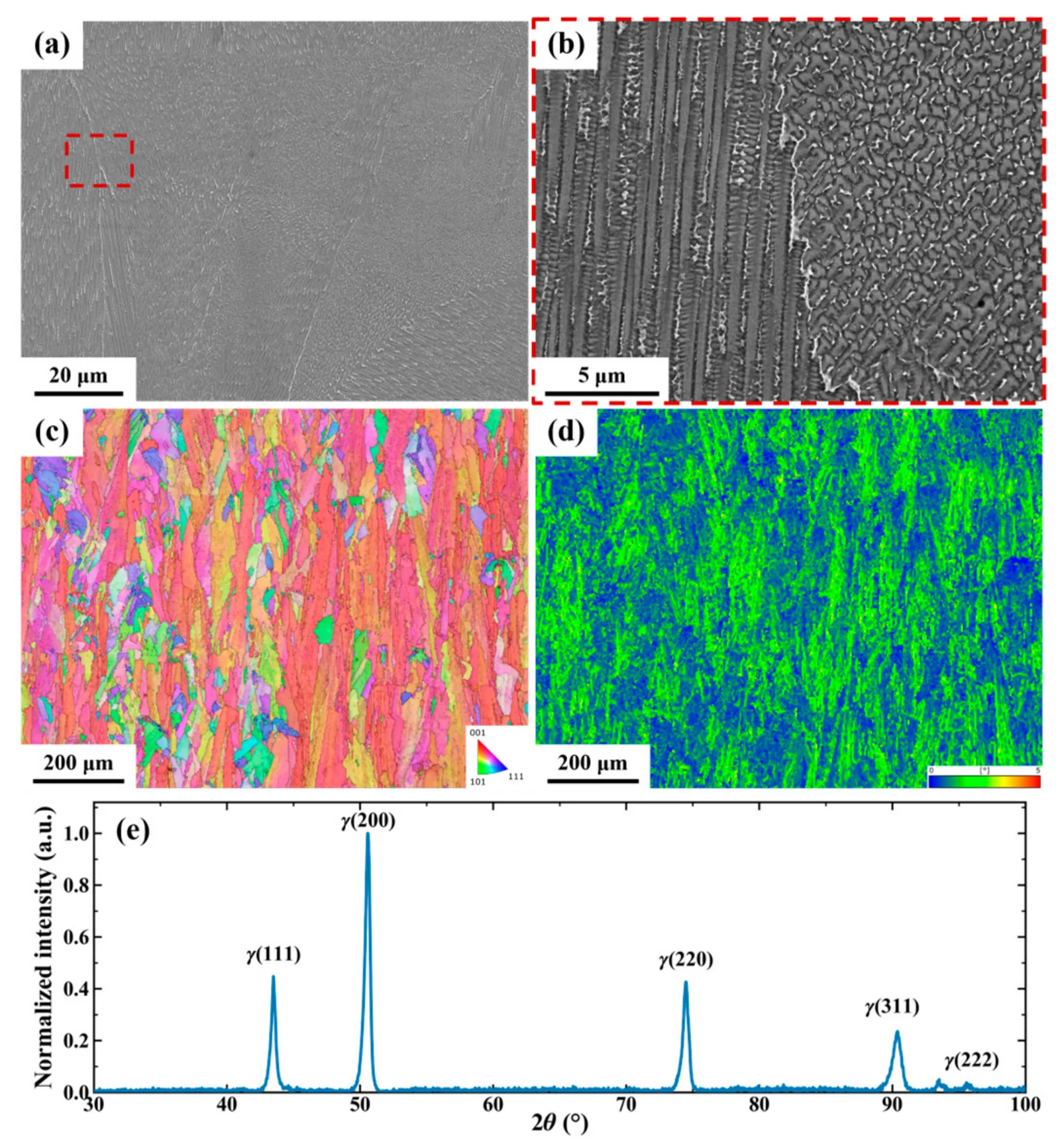
Typical microstructural characteristics of the as-built GH4169 alloy fabricated by selective laser melting (SLM): (**a**) low-magnification scanning electron microscopy (SEM) image; (**b**) high-magnification SEM image of the region enclosed by the red dashed box in (**a**); (**c**) electron backscatter diffraction (EBSD) inverse pole figure (IPF) map; (**d**) kernel average misorientation (KAM) map; and (**e**) representative X-ray diffraction (XRD) pattern of an as-built sample.

Figure 1 shows the typical microstructural characteristics of the as-built GH4169 alloy fabricated by SLM. As shown in Figure 1a, distinct melt-pool overlap boundaries were observed in the low-magnification SEM image, and columnar/cellular solidification structures growing along the direction of the melt-pool boundaries were present in local regions. The enlarged image in Figure 1b shows that the grain interiors were mainly composed of cellular substructures, with variations in cell size, morphology, and alignment among different regions. The cell-wall regions exhibited relatively high electron contrast and contained fine secondary phases with continuous or discontinuous distributions. Some of these secondary phases were interconnected along the cell walls, forming chain-like or locally network-like structures, consistent with pronounced solute segregation associated with rapid solidification. The EBSD inverse pole figure map in Figure 1c shows that the as-built grains were predominantly columnar and elongated along the build direction, accompanied by a small number of fine grains. The KAM map in Figure 1d shows that regions with relatively high local misorientation were mainly distributed at grain boundaries and within some banded intragranular regions. Overall, the as-built GH4169 alloy exhibited melt-pool structures, build-direction-oriented columnar grains, cellular substructures, and secondary phases distributed along the cell walls. The representative XRD pattern of an as-built sample (Figure 1e) was dominated by face-centered cubic (FCC) γ-matrix reflections and was used as qualitative complementary evidence for phase comparison.

**Figure 2:**
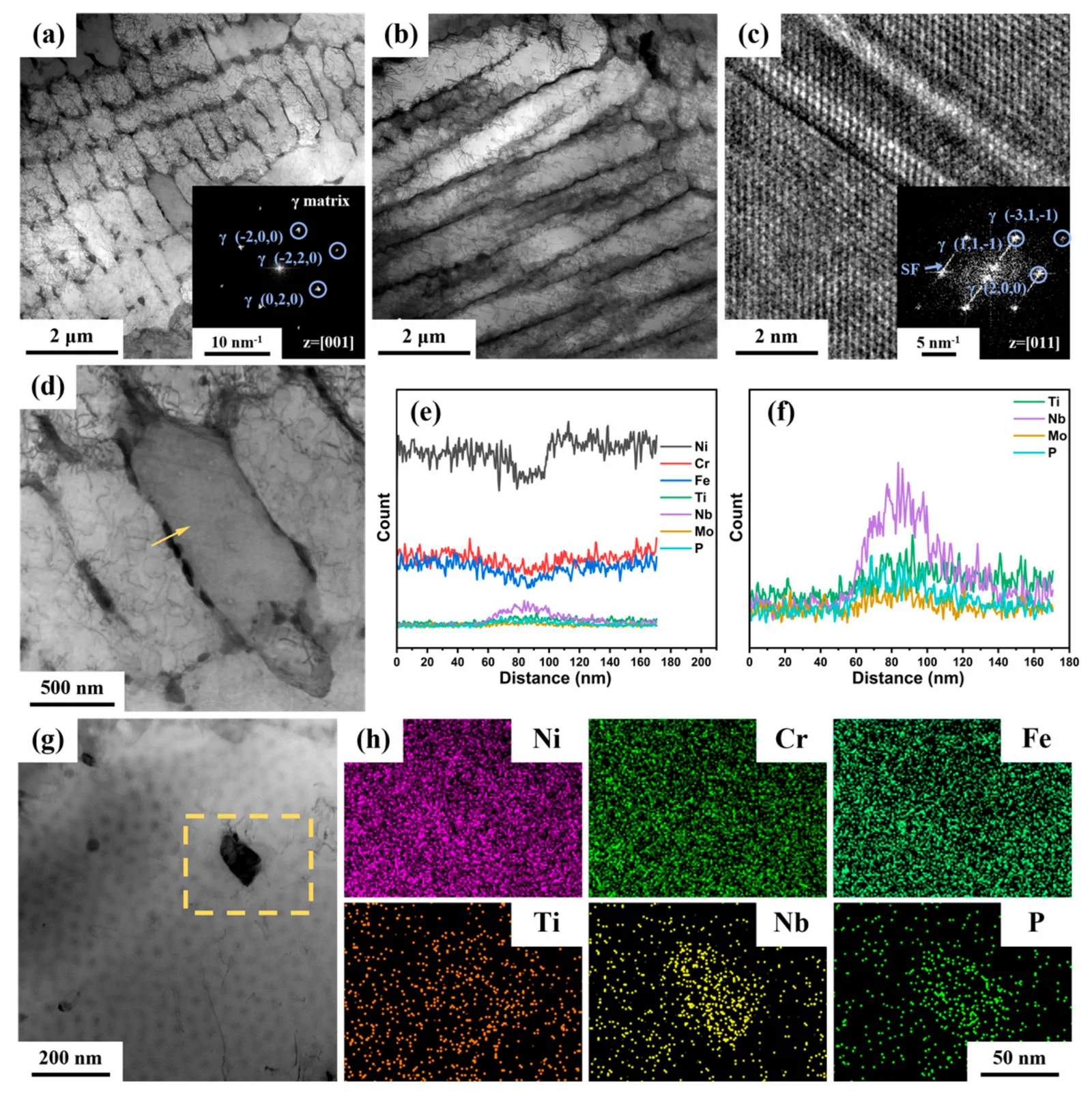
Transmission electron microscopy (TEM) microstructure and elemental distributions of the as-built GH4169 alloy fabricated by SLM: (**a**,**b**) TEM images of the cellular substructures, with the inset in (**a**) showing a representative selected-area electron diffraction (SAED) pattern obtained along the [001] zone axis of the γ matrix; (**c**) high-resolution transmission electron microscopy (HRTEM) image of the stacking faults, with the inset showing the fast Fourier transform (FFT) pattern indexed along the [011] zone axis of the γ matrix; (**d**) TEM image of a cell-wall region and the energy-dispersive X-ray spectroscopy (EDS) line-scan path indicated by the arrow; (**e**) EDS line-scan profiles of Ni, Cr, Fe, Ti, Nb, Mo, and P; (**f**) enlarged line-scan profiles of Ti, Nb, Mo, and P; (**g**) scanning transmission electron microscopy (STEM) image of a particulate secondary phase near the cellular structure and the dashed box indicating the region selected for EDS mapping; and (**h**) STEM-EDS elemental maps of Ni, Cr, Fe, Ti, Nb, and P.

Figure 2 further shows the TEM microstructure and local elemental distributions of the as-built GH4169 alloy. As shown in Figure 2a,b, numerous fine cellular substructures were present within the γ matrix. The cell walls exhibited darker contrast and were interconnected to form continuous or semi-continuous networks, whereas the contrast within the cell interiors was relatively uniform. The SAED pattern shown in the inset of Figure 2a could be indexed along the [001] zone axis of the γ phase, indicating that the matrix of the as-built alloy had an FCC γ structure. In addition to the cellular substructures, mutually parallel band-like lattice contrast is observed in Figure 2c. The corresponding fast Fourier transform (FFT) pattern exhibited streaking near the diffraction spots, indicating the presence of stacking faults in the γ matrix. Figure 2d–f further reveal the elemental distributions across the cell-wall regions. The EDS line-scan results show that the Nb signal increased markedly as the scan crossed a cell wall, whereas the Ni, Cr, and Fe signals decreased. Ti and P also exhibited a certain degree of enrichment, whereas the variation in Mo was relatively weak. These results indicate pronounced solute segregation at the cell walls. Figure 2g,h show a particulate secondary phase located near the cellular structure and its corresponding elemental distributions. The particle exhibited pronounced enrichment of Nb and P, accompanied by a decrease in the Ni signal. Based on its morphology and elemental characteristics, the particle was identified as an Nb-rich secondary phase and was tentatively considered to be a Laves-phase particle.

### 3.2. Microstructural Evolution of the Heat-Treated GH4169 Alloy

**Figure 3:**
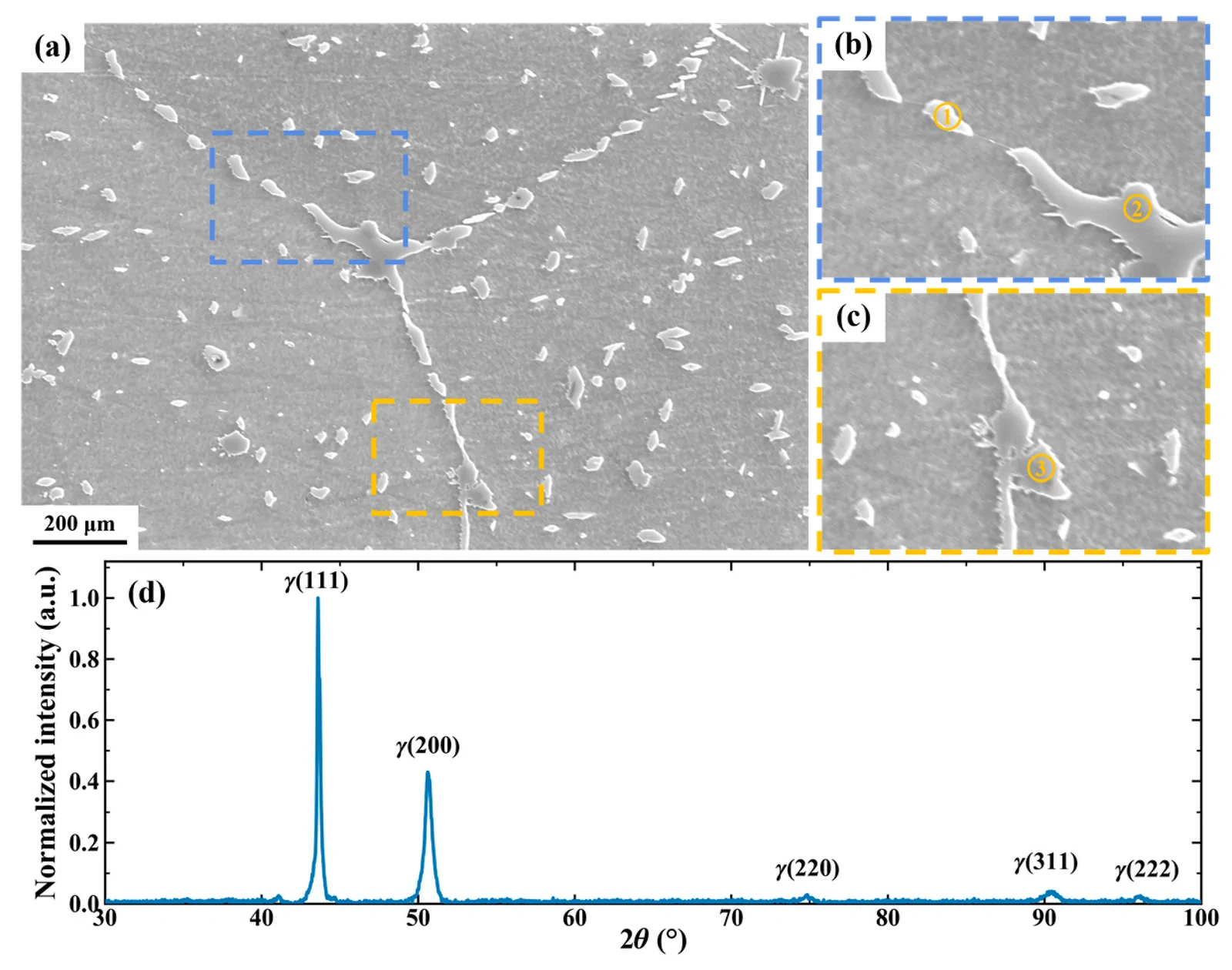
Characteristics of the grain-boundary secondary phases in the heat-treated GH4169 alloy fabricated by SLM: (**a**) SEM image of the grain-boundary secondary phases; (**b**,**c**) enlarged images of the regions enclosed by the blue and yellow dashed boxes in (**a**), respectively; and (**d**) representative XRD pattern of a heat-treated sample. The EDS analysis results for Points 1–3 are presented in Table 1.

Figure 3 shows the characteristics of the grain-boundary secondary phases in the heat-treated GH4169 alloy fabricated by SLM. As shown in Figure 3a, the cellular substructures present in the as-built condition had largely disappeared after heat treatment, and the grain boundaries were clearly distinguishable. Bright-contrast secondary phases were mainly distributed at grain boundaries and grain-boundary junctions. These secondary phases exhibited different morphological characteristics, including particulate or short rod-like precipitates distributed along the grain boundaries and relatively large, irregular blocky particles. Based on Figure 3b,c and the EDS point-analysis results presented in Table 1, the secondary phase at Point 1 contained relatively high concentrations of Ni and Nb, corresponding to 47.65 wt.% Ni and 13.54 wt.% Nb. Based on its combined morphological and compositional characteristics, this phase was tentatively identified as the δ phase. The particle at Point 2 contained relatively high concentrations of Nb and Mo, corresponding to 32.87 wt.% Nb and 3.79 wt.% Mo. Considering its coarse, irregular morphology together with its elemental composition, this particle was tentatively identified as residual Laves phase. The particle at Point 3 was enriched in Nb, C, and Ti, with contents of 29.43 wt.% Nb, 7.73 wt.% C, and 3.24 wt.% Ti, respectively. Based on its compositional and morphological characteristics, the particle was tentatively identified as an MC-type carbide. The representative XRD pattern of a heat-treated sample (Figure 3d) remained dominated by FCC γ-matrix reflections. Because the minor secondary phases have low volume fractions and weak and/or overlapping diffraction peaks, the XRD pattern is interpreted qualitatively, while TEM/SAED provides the principal crystallographic evidence for the secondary phases.

**Figure 4:**
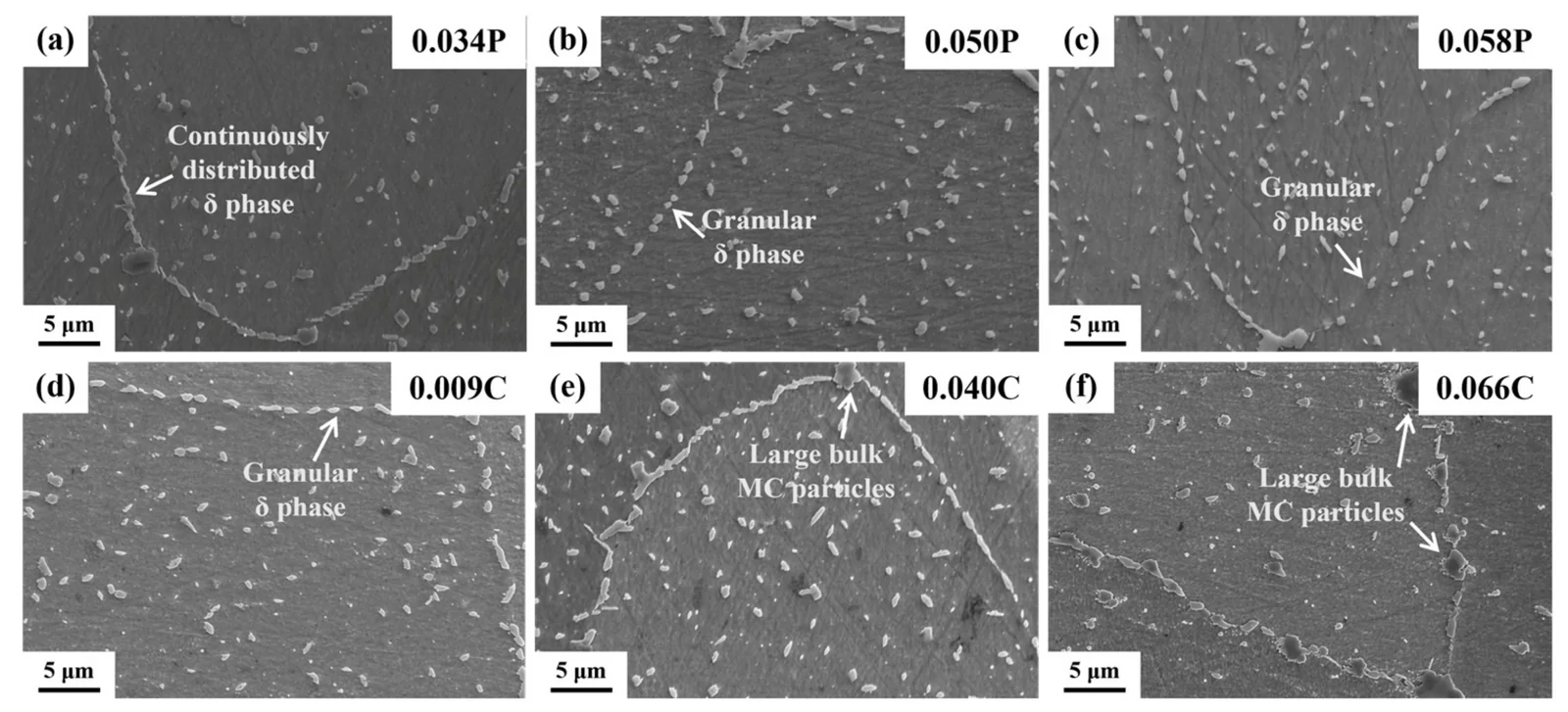
Morphologies of grain-boundary δ phase and MC-type carbides in the heat-treated GH4169 alloys fabricated by SLM with different P and C contents: (**a**) 0.034P; (**b**) 0.050P; (**c**) 0.058P; (**d**) 0.009C; (**e**) 0.040C; and (**f**) 0.066C.

Figure 4 shows the morphologies of the grain-boundary secondary phases in the heat-treated GH4169 alloys fabricated by SLM with different P and C contents. Based on the morphological and EDS characteristics presented in Figure 3, the particulate or short rod-like precipitates distributed along the grain boundaries were interpreted primarily as the δ phase, whereas some of the relatively large, irregular particles were interpreted as MC-type carbides. As shown in Figure 4a–c, δ-phase precipitates were observed at the grain boundaries of all specimens with different P contents. The 0.034P specimen contained a relatively large amount of δ phase, and some particles were interconnected along the grain boundaries, forming a relatively continuous chain-like distribution. As the P content increased to 0.050 and 0.058 wt.%, the amount of grain-boundary δ phase gradually decreased, the interparticle spacing increased, and the distribution progressively changed from a continuous chain-like morphology to semi-continuous and locally discrete distributions. In comparison, variations in C content had a more pronounced effect on the morphology of the grain-boundary secondary phases, as shown in Figure 4d–f. The grain-boundary secondary phases in the 0.009C specimen mainly consisted of fine particulate δ phase, together with a small number of isolated particles interpreted as MC-type carbides. As the C content increased, an increasing number of coarse, blocky MC-type carbides appeared at grain boundaries and grain-boundary junctions, whereas the amount of δ phase gradually decreased. Pronounced coarsening and local agglomeration of MC-type carbides were observed in the 0.066C specimen, while the δ phase exhibited a more dispersed distribution.

**Figure 5:**
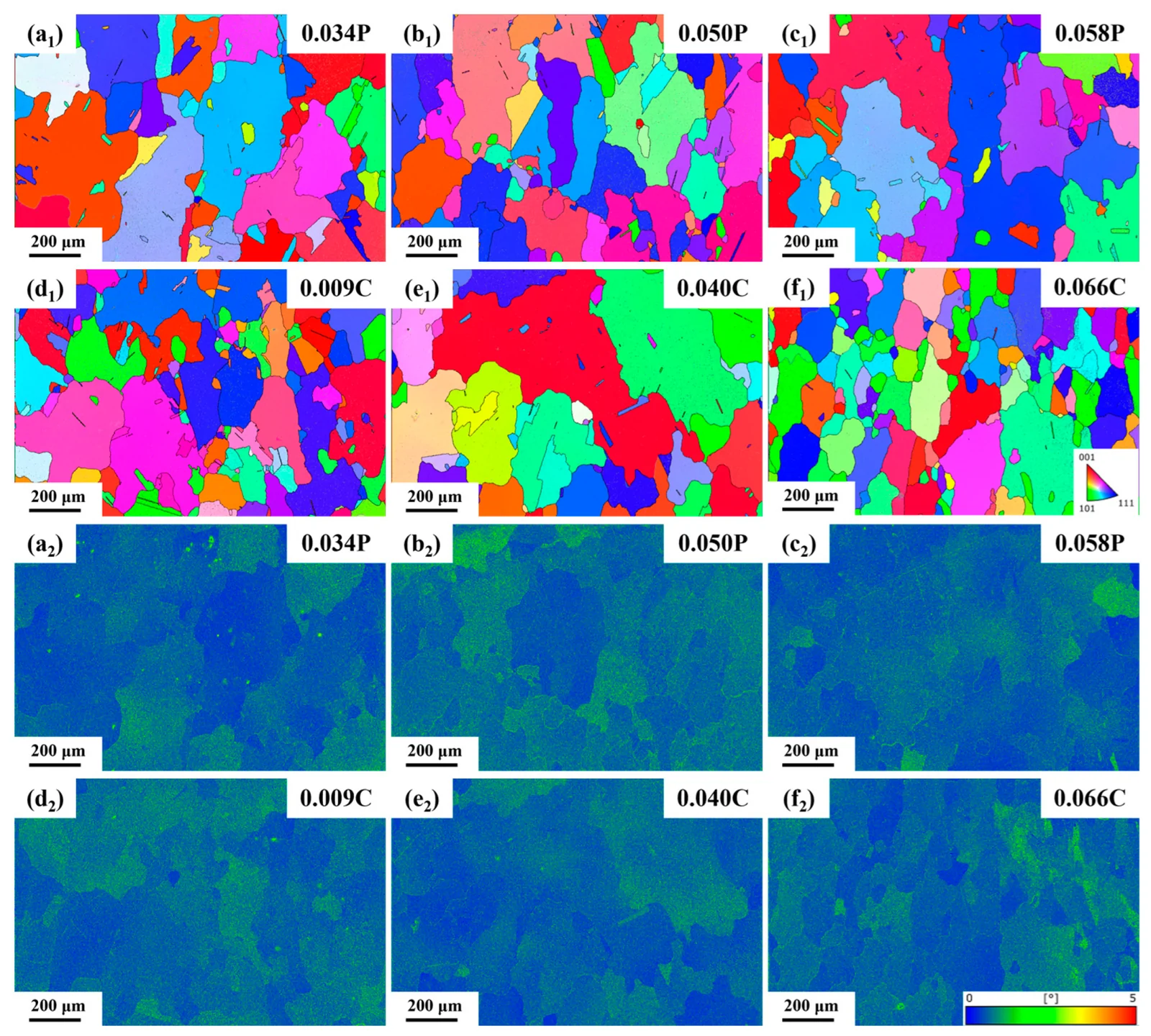
Grain orientations and local misorientation distributions of the heat-treated GH4169 alloys fabricated by SLM with different P and C contents: (**a_1_**–**c_1_**) IPF maps of the 0.034P, 0.050P, and 0.058P specimens; (**d_1_**–**f_1_**) IPF maps of the 0.009C, 0.040C, and 0.066C specimens; and (**a_2_**–**f_2_**) KAM maps of the corresponding specimens.

Figure 5 further shows the grain orientations and local misorientation distributions of the specimens with different P and C contents in the heat-treated condition. As shown in Figure 5(a_1_–f_1_), the grains predominantly exhibited irregular polygonal or near-equiaxed morphologies, although elongated grains remained locally. Across both the P and C series, the overall grain morphologies were broadly comparable, and the present EBSD observations do not show a clear, systematic, or monotonic composition-dependent grain-size change. The corresponding KAM maps in Figure 5(a_2_–f_2_) show that all specimens were dominated by regions with low KAM values. Regions with relatively high KAM values were mainly distributed near the grain boundaries and within some grain interiors, indicating that the heat-treated alloys generally exhibited relatively low local misorientation. Local differences in grain shape and KAM distribution were observed among individual specimens, but these differences did not establish a systematic composition-dependent grain-size trend.

**Figure 6:**
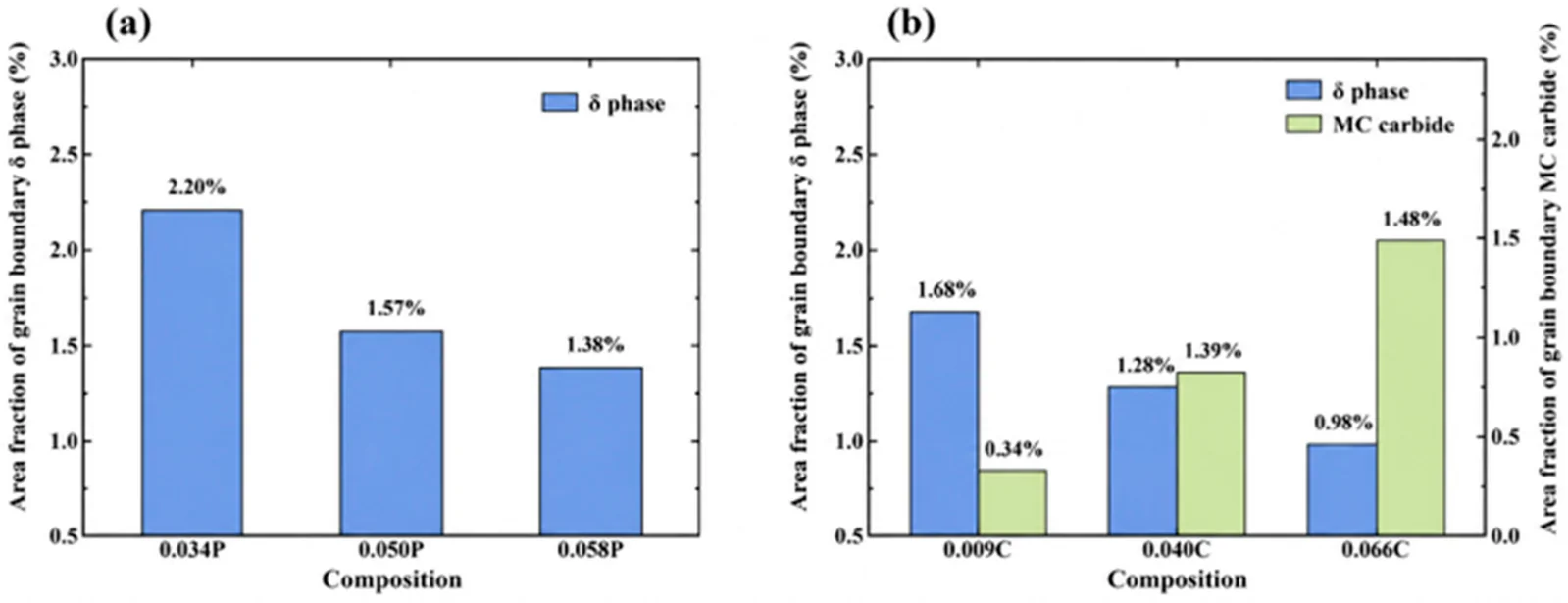
Area fractions of grain-boundary secondary phases in the heat-treated GH4169 alloys fabricated by SLM with different P and C contents: (**a**) area fraction of the grain-boundary δ phase in the specimens with different P contents; and (**b**) area fractions of the grain-boundary δ phase and MC-type carbides in the specimens with different C contents.

Figure 6 presents the statistical results for the area fractions of the grain-boundary secondary phases in the heat-treated specimens with different P and C contents. As shown in Figure 6a, as the P content increased from 0.034 wt.% to 0.050 and 0.058 wt.%, the area fraction of the δ phase decreased successively from 2.20% to 1.57% and 1.38%, consistent with the reductions in the amount and continuity of the δ phase observed in Figure 4. The δ phase and MC-type carbides exhibited different variation trends among the specimens with different C contents, as shown in Figure 6b. As the C content increased from 0.009 wt.% to 0.040 and 0.066 wt.%, the area fraction of the δ phase decreased from 1.68% to 1.28% and 0.98%, whereas the area fraction of MC-type carbides increased from 0.34% to 0.80% and 1.48%, respectively. Consistent with the qualitative EBSD observations in Figure 5, the present data do not support a clear systematic composition-dependent grain-size change.

### 3.3. High-Temperature Tensile Properties and Fracture Morphology

**Figure 7:**
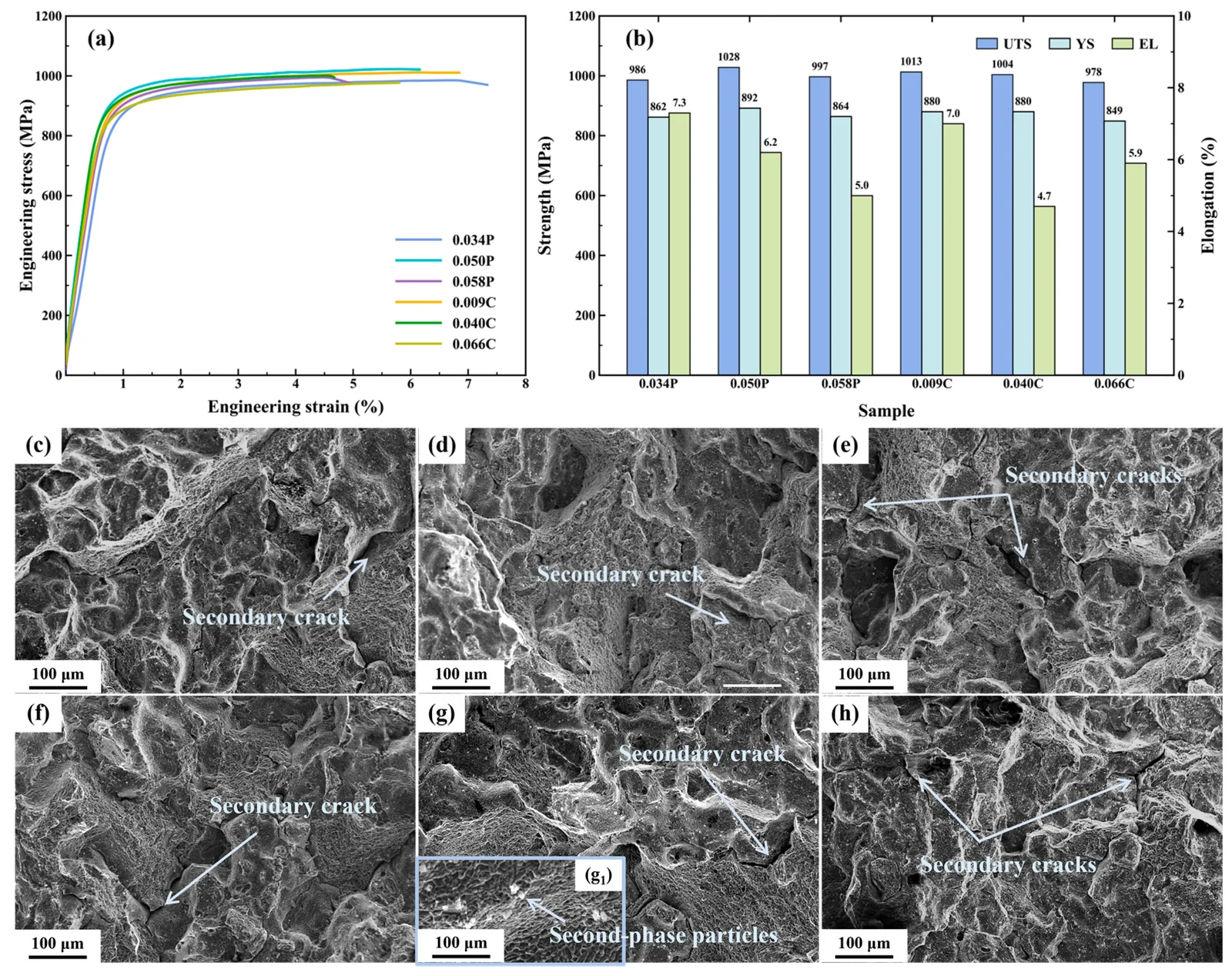
Tensile properties and fracture morphologies of the heat-treated SLM-fabricated GH4169 alloys with different P and C contents at 650 °C: (**a**) engineering stress–engineering strain curves; (**b**) ultimate tensile strength (UTS), yield strength (YS), and elongation to fracture (EL); (**c**–**h**) overview fracture-surface SEM images of the 0.034P, 0.050P, 0.058P, 0.009C, 0.040C, and 0.066C specimens, respectively (nominal magnification 300×; 100 μm scale bars); and (**g_1_**) enlarged image of a local region in (**g**), showing secondary-phase particles.

Figure 7 shows the tensile properties and fracture morphologies of the heat-treated SLM-fabricated GH4169 alloys with different P and C contents at 650 °C. As shown in Figure 7a,b, variations in P content had a pronounced effect on the high-temperature tensile properties. When the P content increased from 0.034 to 0.050 wt.%, the yield strength increased from 862 to 892 MPa, and the ultimate tensile strength increased from 986 to 1028 MPa, whereas the elongation to fracture decreased from 7.3% to 6.2%. When the P content was further increased to 0.058 wt.%, the yield strength and ultimate tensile strength decreased to 864 and 997 MPa, respectively, while the elongation to fracture further decreased to 5.0%.

Variations in C content also affected the high-temperature tensile properties. As the C content increased from 0.009 to 0.040 wt.%, the yield strength remained essentially unchanged at approximately 880 MPa, the ultimate tensile strength decreased from 1013 to 1004 MPa, and the elongation to fracture decreased from 7.0% to 4.7%. When the C content was further increased to 0.066 wt.%, the yield strength and ultimate tensile strength decreased to 849 and 978 MPa, respectively, whereas the elongation to fracture increased to 5.9%. Among the C-series specimens, the 0.009C specimen exhibited a favorable high-temperature strength–ductility balance.

Figure 7c–h show the high-temperature tensile fracture morphologies of the different specimens. The fracture surfaces of all specimens exhibited mixed fracture characteristics, with the coexistence of dimpled regions and relatively flat fracture regions, accompanied by secondary cracks to varying degrees. Secondary-phase particles were observed within or near some dimples, as shown in Figure 7(g_1_), suggesting that these particles participated in the high-temperature deformation and fracture processes.

### 3.4. TEM Characterization of Heat-Treated Microstructure

**Figure 8:**
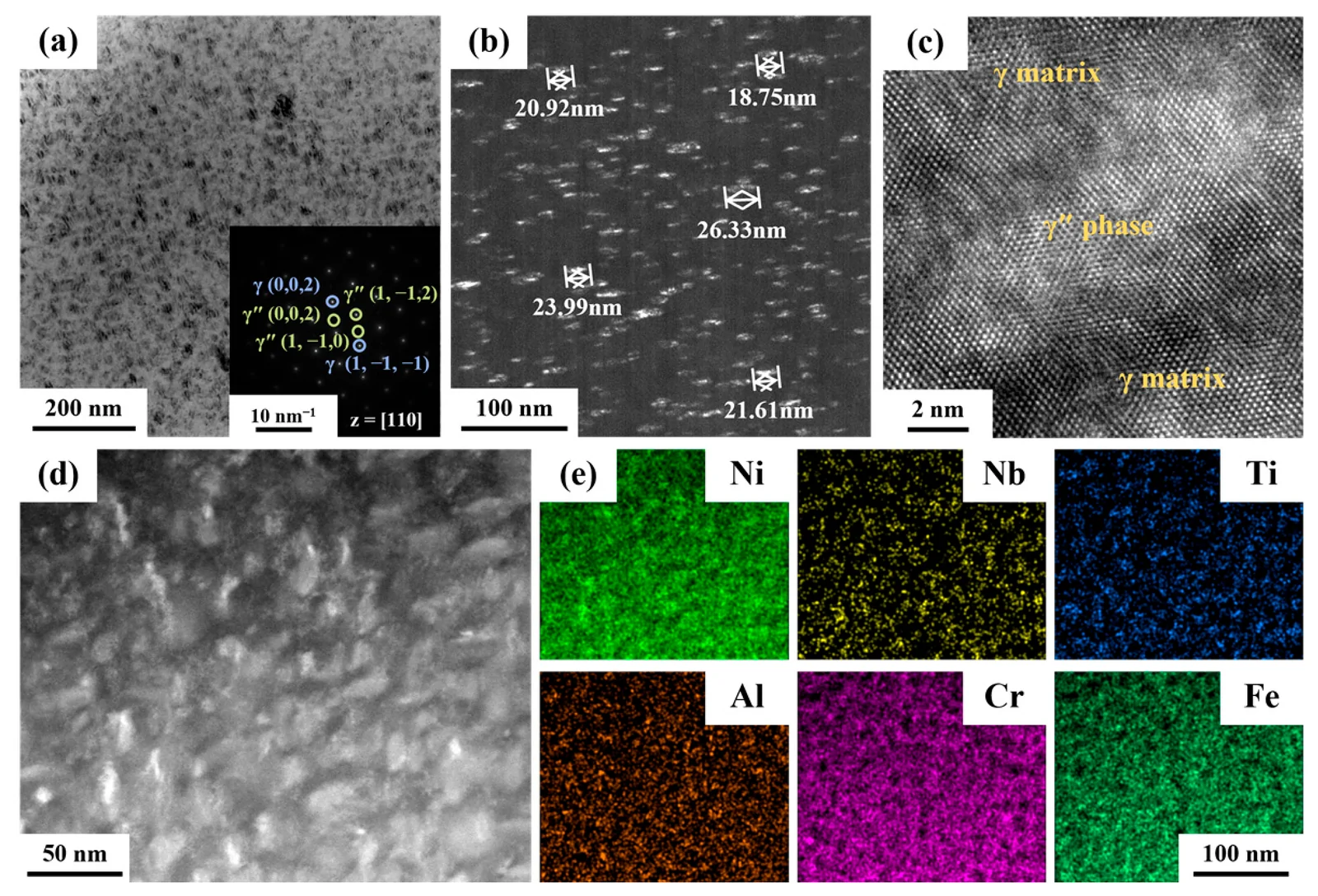
TEM characterization of nanoscale strengthening precipitates in the heat-treated SLM-fabricated GH4169 alloy: (**a**) bright-field TEM image of the nanoscale precipitates in the γ matrix, with the inset showing an SAED pattern obtained along the [110] zone axis of the γ matrix; (**b**) dark-field TEM image of the γ″ phase and the major-axis lengths of selected particles; (**c**) HRTEM image of the γ″ phase and its interface with the γ matrix; (**d**) STEM image of the nanoscale precipitates; and (**e**) STEM-EDS elemental maps of Ni, Nb, Ti, Al, Cr, and Fe. Fine Ti/Al-rich particles were tentatively attributed to γ′ based on morphology and composition.

Figure 8 shows the morphology, crystallographic evidence, and elemental-distribution characteristics of nanoscale precipitates in the heat-treated SLM-fabricated GH4169 alloy. As shown in Figure 8a, numerous nanoscale precipitates were dispersed throughout the γ matrix, with no evident coarse particles or agglomeration. In addition to the diffraction spots of the γ matrix, superlattice reflections associated with the γ″ phase were observed in the corresponding SAED pattern, indicating the formation of the γ″ phase with a D0_22_ structure after heat treatment.

The dark-field image in Figure 8b shows that the γ″ precipitates mainly exhibited fine elliptical or short rod-like morphologies. The precipitates measured in this representative image had an average major-axis length of approximately 22.32 nm. The HRTEM image in Figure 8c shows a distinct interface between the precipitate and the γ matrix, together with a relatively good lattice match. The STEM-EDS results in Figure 8d,e show Nb enrichment in some elongated precipitate regions, consistent with the compositional characteristics of the γ″ phase. Some fine particles exhibited Ti and Al enrichment and were tentatively identified as γ′ precipitates based on their morphological and compositional characteristics; no equivalent crystallographic confirmation of γ′ was obtained in the present dataset.

**Figure 9:**
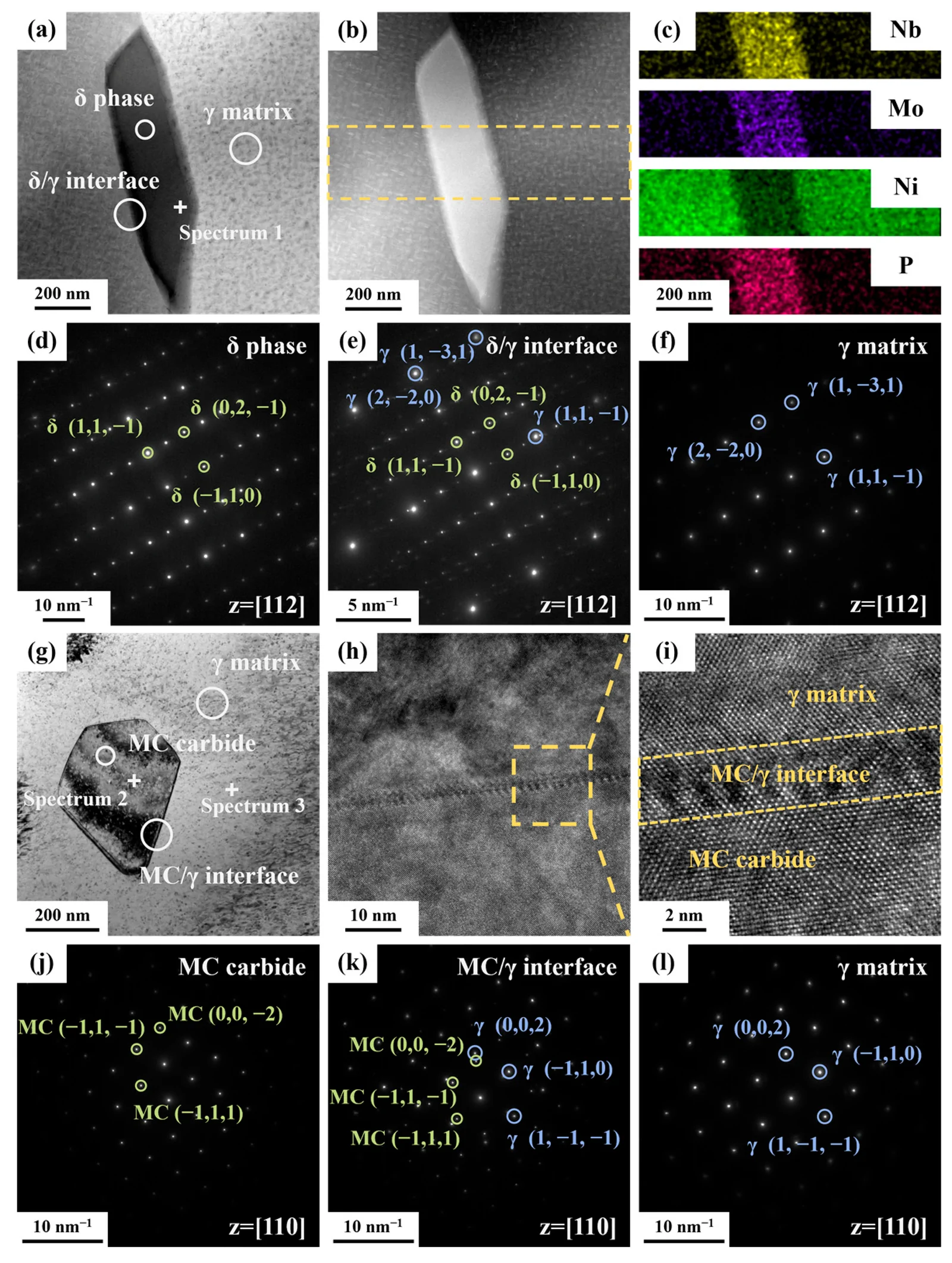
TEM characterization of the δ phase and an Nb-rich MC-type carbide in the heat-treated SLM-fabricated GH4169 alloy: (**a**) bright-field TEM image of the δ phase and the locations selected for SAED and EDS point analysis; (**b**) STEM image of the δ phase and the dashed box indicating the region selected for EDS mapping; (**c**) STEM-EDS elemental maps of Nb, Mo, Ni, and P; (**d**–**f**) SAED patterns of the δ phase, δ/γ interface, and γ matrix, respectively; (**g**) bright-field TEM image of the Nb-rich MC-type carbide and the locations selected for SAED and EDS point analysis; (**h**,**i**) HRTEM image of the MC/γ interface and a locally enlarged image; and (**j**–**l**) SAED patterns of the MC-type carbide, MC/γ interface, and γ matrix, respectively.

Figure 9 further presents the TEM characterization results for the δ phase and an Nb-rich MC-type carbide in the heat-treated alloy. Figure 9a–f show the characterization results for the δ phase. As shown in Figure 9a, the precipitate exhibited a lath-like morphology and had a distinct interface with the γ matrix. The SAED patterns acquired from the precipitate, the δ/γ interface, and the γ matrix were indexed as the δ phase, the superposition of δ- and γ-phase reflections, and the γ phase, respectively, confirming that the precipitate was the δ phase. The STEM-EDS results show pronounced Nb enrichment in the δ-phase region. According to the point-analysis results in Table 2, the Nb and Mo contents at Point 1 were 24.09 and 10.51 wt.%, respectively, consistent with the compositional characteristics of the δ phase.

Figure 9g–l show the TEM characterization results for the MC-type carbide. The particle exhibited an irregular polygonal morphology and a distinct particle/matrix interface. The SAED results confirmed that the particle had an MC-type carbide crystal structure. The TEM-EDS point-analysis results show that the particle was enriched in Nb, Ti, and C, with contents of 67.78, 9.57, and 7.40 wt.%, respectively. These results identify the particle as an Nb-rich MC-type carbide containing a certain amount of Ti.

Figure 9h,i further show the interface between the Nb-rich MC-type carbide and the γ matrix. The lattice fringes on both sides of the interface were clearly distinguishable and remained continuous near the interface. No evident pores, amorphous layers, or interfacial separation were observed. In summary, both a lath-like δ phase and an irregular polygonal Nb-rich MC-type carbide were present in the heat-treated alloy. These two phases exhibited distinct differences in morphology, crystal structure, and elemental composition, providing TEM evidence for distinguishing the different grain-boundary secondary phases observed in the preceding SEM images.

## 4. Discussion

SLM melt-pool solidification is generally associated with steep thermal gradients and high cooling rates that promote nonequilibrium microstructural development; however, G, R, and cooling rate were not measured in the present study, so the observed structure is interpreted by comparison with reported IN718 SLM behavior rather than by assigning process-specific thermal values [23,24]. The SEM and TEM results in Figure 1 and Figure 2 show that the as-built GH4169 alloy consisted of columnar grains extending predominantly along the build direction and fine-scale cellular substructures. Similar build-direction-oriented columnar grain morphologies have been reported for SLM-fabricated IN718 [4,25]. In this context, “rapid solidification” refers to the high cooling rate experienced by the transient laser melt pool during solidification rather than to the ambient or substrate temperature. Pronounced solute segregation occurred at the cell walls and was accompanied by the precipitation of Nb-rich secondary phases. Previous studies have reported that limited solute diffusion during SLM solidification causes elements such as Nb, Ti, and Mo to preferentially accumulate in the last-to-solidify liquid and ultimately remain at cell walls or contribute to the formation of Nb-rich Laves-phase particles [4,25]. The present study further showed that P also exhibited a certain degree of enrichment at the cell walls, indicating that P participated in the chemical segregation established during solidification in the as-built microstructure.

After heat treatment, the cellular substructures present in the as-built condition had largely disappeared, and the KAM distributions appeared lower, suggesting relaxation of local orientation gradients and a possible reduction in the associated dislocation density [9,26,27]. Meanwhile, segregation at the cell walls weakened, and some of the Laves phase dissolved, allowing Nb to re-enter the γ matrix, whereas incompletely dissolved Laves phase remained near the grain boundaries, as shown in Figure 3 [10,23,24]. TEM analysis confirmed the γ″ phase, δ phase, and an Nb-rich MC-type carbide, while fine Ti/Al-rich particles without equivalent crystallographic confirmation were tentatively identified as γ′, as shown in Figure 8 and Figure 9. Previous studies have shown that changes in the amount and distribution of the δ phase can affect the mechanical response of IN718, including high-temperature performance and notch sensitivity [28,29]. The remaining Nb-rich phases and strengthening precipitates reflected substantial redistribution of Nb during post-processing. These observations are consistent with recent studies describing the evolution of additively manufactured IN718 during heat treatment [9,23,24,30,31]. This redistribution established a new Nb partitioning state among the γ matrix, γ″ phase, δ phase, MC-type carbides, and residual Laves phase and provided an important basis for the subsequent composition-dependent secondary-phase evolution.

The as-built alloy exhibited build-direction-oriented columnar grains, whereas after heat treatment the grains were predominantly irregular polygonal or near-equiaxed, although elongated grains remained locally. This change suggests that heat treatment reduced the morphological anisotropy inherited from SLM without demonstrating its complete elimination. Because the present EBSD characterization and 650 °C tensile testing were referenced to BD and no matched transverse-direction (TD) dataset was acquired, a quantitative BD–TD mechanical anisotropy cannot be established from the present results. Previous studies have likewise shown that build direction and subsequent heat treatment can influence the directional tensile response of additively manufactured IN718 [32,33].

### 4.1. Effect of P on δ Precipitation Behavior and High-Temperature Tensile Properties

P is a typical grain-boundary-segregating element in GH4169 alloys, and its influence on high-temperature properties is mainly associated with its interfacial segregation behavior. Previous studies have shown that P preferentially segregates to grain boundaries and δ/γ interfaces and affects δ-phase precipitation by altering the local interfacial energy, atomic diffusion behavior, and nucleation conditions of the δ phase [18,20]. Although the P segregation levels at grain boundaries and interfaces were not directly measured in the present study, the TEM results for the as-built condition showed that P was enriched at the cell walls formed during rapid solidification, as shown in Figure 2. This result indicates that the role of P in SLM-fabricated GH4169 alloys was not limited to the heat-treatment stage but was also influenced by the nonequilibrium segregation microstructure established in the as-built condition. Because the solidification conditions differ from those of conventionally cast or wrought GH4169 alloys, this segregation state may influence the subsequent P-related microstructural evolution in SLM-fabricated GH4169 alloys.

As the P content increased, the amount and continuity of the grain-boundary δ phase decreased. The available EBSD observations do not indicate a clear systematic P-dependent grain-size change. Because the P concentration at grain boundaries and δ/γ interfaces was not quantified directly in the present study, the mechanism is interpreted with reference to previous reports: interfacial P segregation may modify the local interfacial energy, atomic diffusion, and δ-phase nucleation conditions, thereby reducing the tendency for continuous δ-phase precipitation [18,20].

The 0.050P alloy combined reduced δ-phase continuity with the highest yield and ultimate tensile strengths at 650 °C. When the P content was further increased to 0.058 wt.%, both strength and ductility decreased, indicating that the beneficial effect is limited to an appropriate P range rather than increasing monotonically with P content.

### 4.2. Effect of C on MC/δ Competitive Precipitation Behavior and High-TemperatureTensile Properties

For the C series, increasing C promoted the formation and coarsening of MC-type carbides while the amount of δ phase decreased. TEM identified an Nb-rich MC-type carbide containing a certain amount of Ti at the grain boundaries in the heat-treated condition, as shown in Figure 9. Because both MC-type carbides and the δ phase are Nb-rich phases and consume Nb, their opposing variation trends are consistent with redistribution of Nb toward carbide formation, although phase-specific Nb partitioning was not quantified directly in the present study. The microstructural statistics therefore support a C-related shift toward MC-type carbide formation accompanied by reduced δ-phase precipitation.

Re-examination of the EBSD results shows that the overall grain morphology is broadly comparable among the investigated compositions, and the present data do not support a clear, systematic, or monotonic dependence of grain size on P or C content. Solute segregation, changes in the amount, distribution, and coarsening of secondary phases, and dissolution or redistribution of Nb-rich secondary phases may influence grain-boundary migration during post-processing. However, the relative contributions of solute drag, phase dissolution, and secondary-phase pinning cannot be quantified from the present results. Grain size is therefore treated as a secondary microstructural factor rather than as a confirmed P- or C-controlled mechanism, and no unique mechanism of grain-size evolution is assigned.

The tensile results at 650 °C further reflected this secondary-phase evolution. The 0.009C specimen exhibited the best strength–ductility balance. As the C content increased, the high-temperature strength generally decreased, whereas the ductility exhibited a non-monotonic variation, as shown in Figure 7. The evolution and redistribution of the grain-boundary secondary phases may modify local grain-boundary constraint and deformation compatibility, which is consistent with the generally reduced tensile strength and the non-monotonic ductility observed at 650 °C. Because phase-specific Nb partitioning and the individual contributions of carbide coarsening and δ-phase reduction were not quantified directly, no unique C-dependent deformation mechanism is assigned.

**Figure 10:**
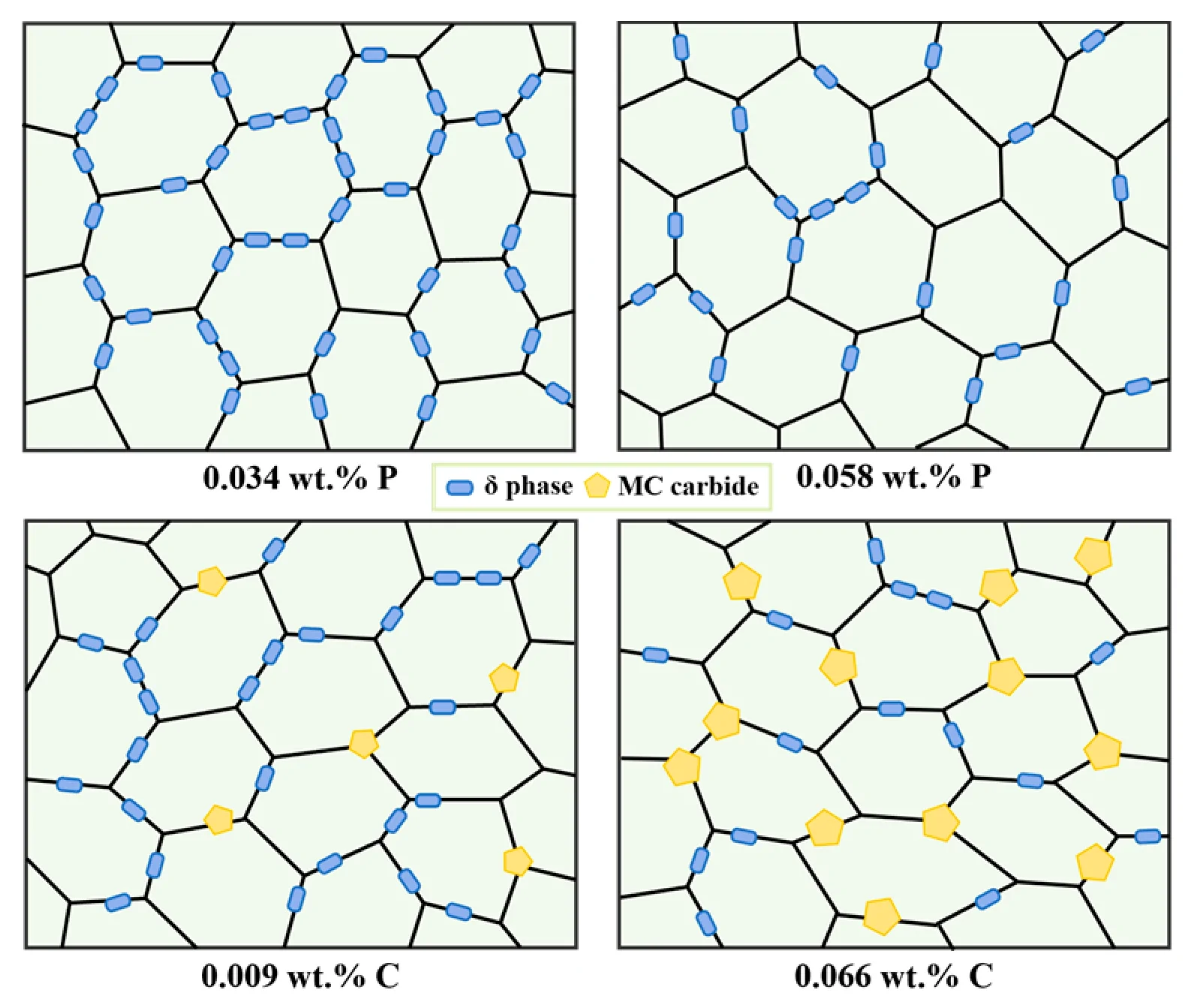
Schematic summary of the P- and C-related secondary-phase evolution and its proposed relationship with tensile behavior at 650 °C in SLM-fabricated GH4169 alloys.

Figure 10 summarizes the experimentally observed P- and C-related secondary-phase evolution and its proposed relationship with tensile behavior at 650 °C. For the P series, increasing P is associated mainly with a decrease in the amount and continuity of the grain-boundary δ phase. For the C series, increasing C promotes a higher fraction and coarsening of MC-type carbides and reduces the δ-phase fraction. The available EBSD observations do not support a clear systematic composition-dependent grain-size change; accordingly, the schematic depicts broadly comparable grain scale across compositions and does not use grain size as an intermediate mechanism. The proposed links involving interfacial P effects and Nb redistribution are mechanistic interpretations based on the present observations and the literature, rather than direct measurements of interfacial energy or phase-specific Nb partitioning.

The present study shows that the nonequilibrium segregation microstructure formed during SLM provided the initial chemical and microstructural conditions that influenced Nb redistribution during subsequent heat treatment. Consequently, P and C affected microstructural evolution through distinctly different pathways. P was associated mainly with changes in the amount and distribution state of the grain-boundary δ phase, whereas C was associated mainly with redistribution of Nb among MC-type carbides, the δ phase, and the γ matrix and with corresponding changes in grain-boundary secondary-phase distribution. These secondary-phase changes were in turn associated with different tensile responses at 650 °C. Therefore, the optimization of trace elements in SLM-fabricated GH4169 alloys should consider the initial segregation state produced during solidification and subsequent elemental redistribution during heat treatment. Within the compositional range investigated in this study, the alloy containing 0.050 wt.% P exhibited the highest high-temperature strength, whereas the alloy containing 0.009 wt.% C exhibited the best strength–ductility balance, providing experimental evidence for the microalloying design of SLM-fabricated GH4169 alloys.

## 5. Conclusions

The representative as-built microstructure was characterized, and the effects of varying trace P and C contents on the heat-treated microstructure, and tensile properties at 650 °C of SLM-fabricated GH4169 alloys were systematically investigated. The main conclusions are as follows:

(1) The as-built GH4169 alloy was mainly composed of columnar grains growing along the build direction and cellular substructures. Pronounced elemental segregation occurred at the cell walls, with Nb exhibiting the most prominent enrichment, accompanied by local enrichment of Ti, Mo, and P and the precipitation of Nb-rich Laves-phase particles. After heat treatment, the cellular substructures present in the as-built condition largely disappeared, nanoscale γ″ strengthening precipitates formed in the γ matrix, and fine Ti/Al-rich particles were tentatively identified as γ′. The δ phase, an Nb-rich MC-type carbide, and a small amount of residual Laves phase were also present at the grain boundaries. These changes resulted in the transformation of the rapidly solidified nonequilibrium microstructure into a precipitate-containing heat-treated microstructure.
(2) As the P content increased from 0.034 to 0.058 wt.%, the area fraction of the grain-boundary δ phase decreased from 2.20% to 1.38%, and its distribution gradually changed from relatively continuous chains of particles to semi-continuous and locally discrete distributions. The available EBSD observations did not reveal a clear systematic P-dependent grain-size change. The tensile strength at 650 °C initially increased and subsequently decreased with increasing P content, whereas the elongation to fracture continuously decreased. Variations in P content were associated mainly with the precipitation behavior of the grain-boundary δ phase and the high-temperature strength–ductility balance.
(3) As the C content increased from 0.009 to 0.066 wt.%, the area fraction of MC-type carbides increased from 0.34% to 1.48%, whereas the area fraction of the δ phase decreased from 1.68% to 0.98%. These changes were accompanied by the coarsening and local agglomeration of MC-type carbides, while the available EBSD observations did not support a clear monotonic C-dependent grain-size change. The tensile strength at 650 °C generally decreased. Among the C-series specimens, the 0.009C specimen exhibited the best strength–ductility balance, with a yield strength, ultimate tensile strength, and elongation to fracture of 880 MPa, 1013 MPa, and 7.0%, respectively. These results indicate that variations in C content were associated mainly with the evolution of MC-type carbides and the δ phase, as well as the high-temperature mechanical properties.
(4) The Nb segregation generated during solidification in SLM provided the microstructural basis for the evolution of Nb-rich secondary phases during heat treatment. During heat treatment, Nb was redistributed among the γ matrix, γ″ strengthening precipitates, δ phase, Laves phase, and MC-type carbides. Variations in P content were mainly associated with changes in the precipitation behavior of the grain-boundary δ phase, whereas variations in C content were mainly associated with changes in the competition between MC-type carbide precipitation and δ-phase precipitation. The two trace elements affected the heat-treated microstructure and high-temperature mechanical properties at 650 °C through different secondary-phase evolution pathways, providing a reference for the microalloying design and coordinated optimization of the microstructure and properties of SLM-fabricated GH4169 alloys.

## Figures and Tables

**Table 1:** EDS point-analysis results for the grain-boundary secondary phases shown in Figure 3 (wt.%).

Point	Ni	Nb	Cr	Fe	Ti	Mo	C
1	47.65	13.54	17.40	16.50	1.11	2.20	1.59
2	34.35	32.87	12.76	11.91	1.24	3.79	3.09
3	33.97	29.43	13.38	12.25	3.24	0.00	7.73

**Table 2:** TEM-EDS point-analysis results for the selected positions in Figure 9 (wt.%).

Point	Ni	Nb	Cr	Fe	Ti	Mo	C
1	34.75	24.09	12.44	12.56	0.67	10.51	2.46
2	7.29	67.78	2.71	2.55	9.57	0.97	7.40
3	53.50	4.05	18.21	17.08	0.92	2.87	1.58

## Data Availability

The original contributions presented in this study are included in the article. Further inquiries can be directed to the corresponding authors.
